# Practical implementation of COVID-19 patient flags into an antimicrobial stewardship program’s prospective review

**DOI:** 10.1017/ice.2020.133

**Published:** 2020-04-15

**Authors:** Ryan W. Stevens, Lynn Estes, Christina Rivera

**Affiliations:** Department of Pharmacy Services, Mayo Clinic, Rochester, Minnesota

*To the Editor—*In March 2020, a call to action was issued for antimicrobial stewardship programs (ASP) to assist in the SARS-CoV-2/COVID-19 response.^1^ Specific attention was focused on the common ASP infrastructures of prospective audit, existing partnerships with microbiology laboratories, and experience in stewarding medication resources as justification for ASP involvement. We leveraged our existing Enterprise ASP prospective audit platform to contribute to the response. Here we describe the logic and development of COVID-19 ASP flags, which were rapidly operationalized in the enterprise electronic medical record (EMR).

Our prospective audit system utilizes a longstanding, homegrown flagging system that was converted to function within the EMR (Epic Systems, Verona, WI).^2-4^ It generates a patient list based on a series of “rules” with complex logic incorporating medications and order elements, laboratory values, and microbiology, etc. The system also allows for the documentation of actions taken and provider response. Interventions deemed complete can be dismissed (ie, removed), and those requiring follow-up can be deferred for later review.

As the burden of COVID-19 patients began to increase, and amid concerns regarding medication shortages, our ASP needed a mechanism to identify patients with a SARS-CoV-2 polymerase chain reaction (PCR) testing performed and/or patients receiving medications in need of careful stewardship. We elected to incorporate both the PCR result and potential COVID-19 therapies into the rule logic because flagging the medications alone would not filter out non–COVID-19 indications, leading to the addition of low value flags or “noise” into the system. We considered flagging only PCR-positive patients, but this approach would fail to identify pending testing or PCR-negative patients who remained on potentially inappropriate medications. Conversely, incorporation of all ordered PCRs would also have contributed a great deal of noise. We developed a hybrid approach by designing 2 flags that identify opportunities for stewardship of medications and confirm infectious diseases (ID) consultation.

The first rule (ASP COVID-19 rule 1) uses logic that identifies inpatients with a negative SARS-CoV-2 PCR test, collected within the previous 7 days, who also have a medication order that may represent “active therapy” (Table 1). Despite the 2 studies by Gautret et al^5,6^ claiming the benefit of the combination of hydroxychloroquine and azithromycin, we deliberately omitted azithromycin from the “active therapy” list.^5,6^ We recognized that despite significant weaknesses in this literature, providers may still order the combination; however, including azithromycin would have introduced flags for appropriately prescribed azithromycin for non–COVID-19 indications. Additionally, patients prescribed the combination of hydroxychloroquine and azithromycin would be flagged by the hydroxychloroquine order, thus making azithromycin inclusion unnecessary.

**Table 1.** Antimicrobial Stewardship COVID-19 Rule Logic

Name	Criteria	Display
ASP COVID-19 rule 1: negative SARS-COV-2 PCR w/ active drug order (inpatients only)	IF negative COVID-19 PCR in last 7 days **AND** IF active order for 1 of the following: Chloroquine Darunavir/ritonavir Hydroxychloroquine Lopinavir/ritonavir Nitazoxanide Remdesivir Ribavirin Sarilumab Tocilizumab Lenzilumab IVIg THEN fire alert	**Rule Text** Rule name COVID-19 medication that triggered flag SARS-COV-2 test result, date, and time
ASP COVID-19 rule 2: positive SARS-COV-2 PCR or pending lab w/ active drug order (inpatients only)	IF positive COVID-19 PCR in last 7 days **OR** IF pending COVID-19 PCR in last 7 days **AND** IF active order for 1 of the following: Chloroquine Darunavir/ritonavir Hydroxychloroquine Lopinavir/ritonavir Nitazoxanide Remdesivir Ribavirin Sarilumab Tocilizumab Lenzilumab IVIg THEN fire alert	**Rule Text** Rule name COVID-19 medication that triggered flag SARS-COV-2 test result, date, and time

Note. SARS-CoV-2, severe acute respiratory syndrome coronavirus 2; PCR, polymerase chain reaction; COVID-, coronavirus disease 2019; IVIg, intravenous immune globulin.

The second rule (ASP COVID-19 rule 2) is triggered by 2 criteria. The first is an inpatient with a positive PCR result irrespective of active medication orders. Our Enterprise team felt that review of all PCR-positive patients would be important to ensure appropriate involvement of the ID team, consideration for enrollment in clinical trials, and candidacy for off-label therapy. Had we stopped here, we would have realized a gap in the ability to review patients for which tests were ordered and pending. This feature was particularly important for facilities within the enterprise with a slower PCR turnaround time. The second criterion is an inpatient with a pending test, but to limit noise, the rule is only triggered when there is also an “active therapy” order. This criterion prompts the reviewer to use the flag “defer” logic for follow-up of test results so that, if positive, it may be re-reviewed by the ASP team. If the result is negative and therapy order remains active, ASP COVID-19 rule 1 is triggered.

Once either of the rules triggers a review, it is accompanied by text that displays the rule name, the active medication order contributing to the logic, and the date, time, and result of the PCR test.

Developing rules that satisfy the needs of the Enterprise ASP as a whole required consideration of each facility’s typical flag burden, testing availability and turnaround time, availability of ID consultation, and onsite clinical trials. Notably, we are not currently an epicenter of the outbreak, and we recognize that, for facilities experiencing a high volume of COVID-19 hospitalizations, the tools described may not be applicable or may need modification prior to implementation. The landscape of COVID-19 management is rapidly evolving. Therefore, we remain nimble in our ability to add or subtract medications from the “targeted therapy” list, and we understand that as SARS-CoV-2 community prevalence or testing recommendations change, the rules should be modified to produce the highest benefit within limited ASP resources. Another factor that contributed to the success of our design is the availability of an internally developed SARS-CoV-2 PCR test. Facilities desiring to use test results as an element of the logic in their ASP triggers should assure that, regardless of testing location, the result is discretely documented in the EHR.

We recognize that this functionality has limitations, and we anticipate that further challenges may arise. However, our goal is to carefully consider how to leverage existing infrastructure to effectively steward critical medication resources without overburdening the ASP team. We hope that describing our ASP’s efforts empowers others to identify optimal design, critical tasks, and high-value interventions contributing to the identification, triage, and management of COVID-19 patients. For healthcare teams of all kinds, it truly is time for “all hands on deck.”
